# Supplementary material for: Salen-like Chromium and Aluminum Complexes as Catalysts in the Copolymerization of Epoxides with Cyclic Anhydrides for the Synthesis of Polyesters
Source: Int J Mol Sci. 2023 Jun 13;24(12):10052. doi: 10.3390/ijms241210052 (PMC10298366; doi:10.3390/ijms241210052)
Supplement: Supplementary file 1 [file ijms-24-10052-s001.zip › ijms-2408346-supplementary.pdf]

Supporting Material

Salen-like Chromium and Aluminum Complexes as Catalysts in the Copolymerization of Epoxides with Cyclic Anhydrides for the Synthesis of Polyesters

Federica Santulli 1, Ilaria Grimaldi 1 , Daniela Pappalardo 2,* , Marina Lamberti 1 and Mina Mazzeo 1,*

1 Department of Chemistry and Biology "A. Zambelli", University of Salerno, Via Giovanni Paolo II, 132, 84084 Fisciano, Italy; fsantulli@unisa.it (F.S.); ilagrimaldi@unisa.it (I.G.); mlamberti@unisa.it (M.L.)

2 Dipartimento di Scienze e Tecnologie, Università del Sannio, Via de Sanctis snc, 82100 Benevento, Italy

** Correspondence: pappalardo@unisannio.it (D.P.); mmazzeo@unisa.it (M.M.)*

Table of Contents

1. Characterization of Complex Cr-2 .... S2
ESI mass spectrometry S2
UV-visible spectroscopy S2
FT-IR spectroscopy S3
2. Ring-opening Copolymerization of epoxides and anhydrides S4
Ring-opening Copolymerization of propylene oxide (PO) and maleic anhydride (MA) and subsequent isomerization S4
3. NMR characterization S5
4. Gel permeation chromatography analysis (GPC) S8

1. Characterization of Complex Cr-2

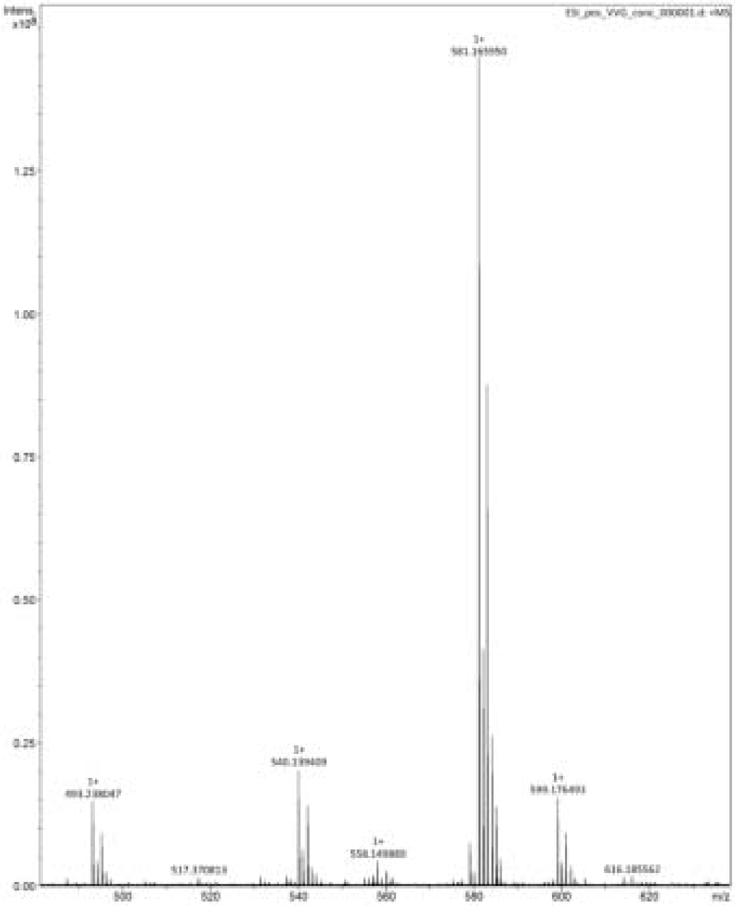

Figure S1. ESI of complex **Cr-2**

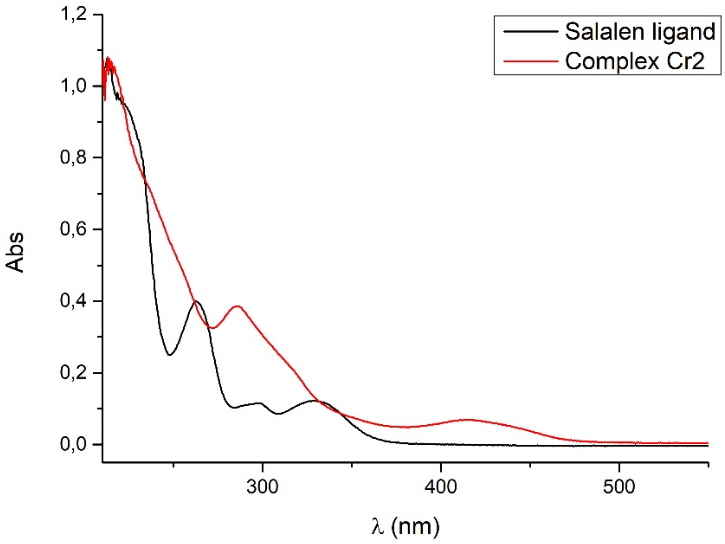

Figure S2. Electronic absorption spectra of salalen ligand and complex **Cr2** in acetonitrile

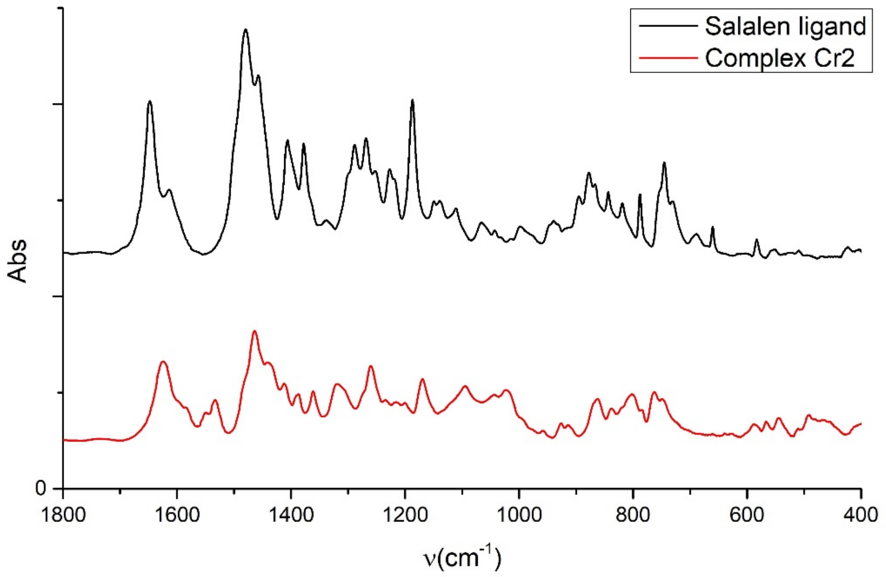

Figure S3. FT-IR spectra of salalen ligand and of the corresponding complex **Cr2**

2. Ring-opening Copolymerization of epoxides and anhydrides

General procedure: Anhydride (PA/SA, 250 equiv.), co-catalyst (DMAP, 2 equiv.), metal complex (1 equiv.) and epoxide (CHO/PO/LO, 1000 equiv.) were added to a 10 mL Schlenk tube equipped with a magnetic stirrer. In some cases, toluene (1 mL) was used as a solvent and 250 equiv. of epoxide were used (see Table 1, entry 1-2). The system was stirred in an oil bath at the required temperature and at different times the reaction was stopped by adding the minimum amount of CH_2Cl_2. A small sample of the crude reaction mixture was used to calculate the conversion by ^1H NMR spectroscopy. The obtained polymer was recovered by filtration and dried in the *vacuum* oven overnight.

Ring-opening Copolymerization of propylene oxide (PO) and maleic anhydride (MA) and subsequent isomerization

General procedure: Maleic anhydride (MA, 200 equiv.), co-catalyst (PPNCl, 1 equiv. when used), and metal complex (Cr2 or Cr4, 1 equiv.) were added to a 10 mL Schlenk tube equipped with a magnetic stirrer. The appropriate solvent (hexane or toluene, 0.50 mL) was added, followed by propylene oxide (PO), added in stoichiometric amounts (200 equiv.) or in excess (1340 equiv., 1 mL). The system was stirred in an oil bath at a temperature of 45°C and at different times the reaction was stopped by adding the minimum amount of CH_2Cl_2. A small sample of the crude reaction mixture was used to calculate the conversion by ^1H NMR spectroscopy. The obtained polymer was recovered by filtration and dried in the *vacuum* oven overnight.

In some cases, diethylamine ($NHEt_2$, 0.1 equiv.) was added directly to the solution at the end of the polymerization to convert poly(propylene) maleate (PPM) to poly(propylene) fumarate (PPF). The solution is stirred at room temperature for 24 hours and the progress of the isomerization is checked by ^1H NMR spectroscopy.

3. NMR characterization of obtained polymers

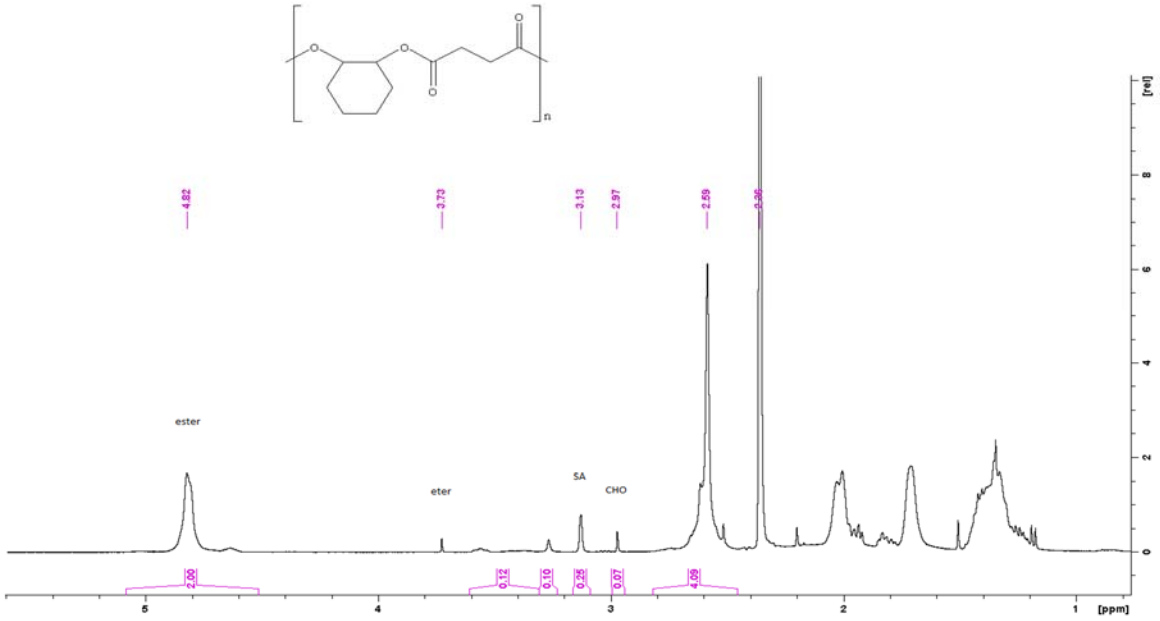

Figure S4. ^{1}H NMR spectrum (300 MHz, toluene, 298K) of CHO/SA copolymer

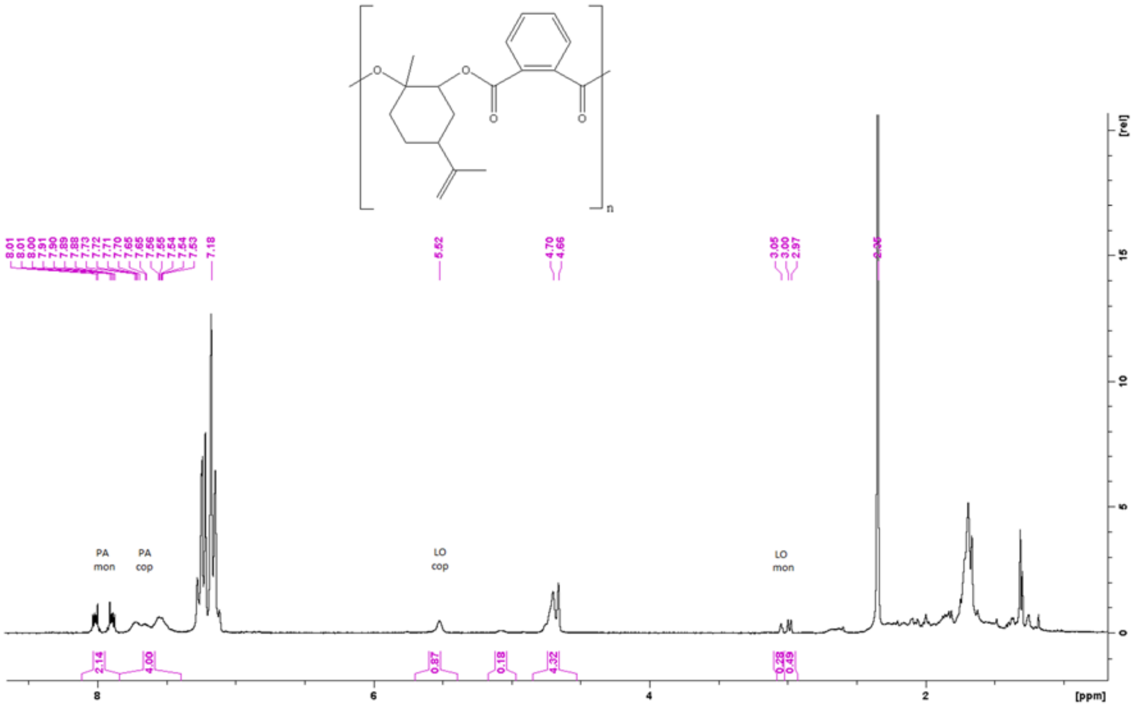

Figure S5. ^{1}H NMR spectrum (300 MHz, toluene, 298K) of LO/PA copolymer

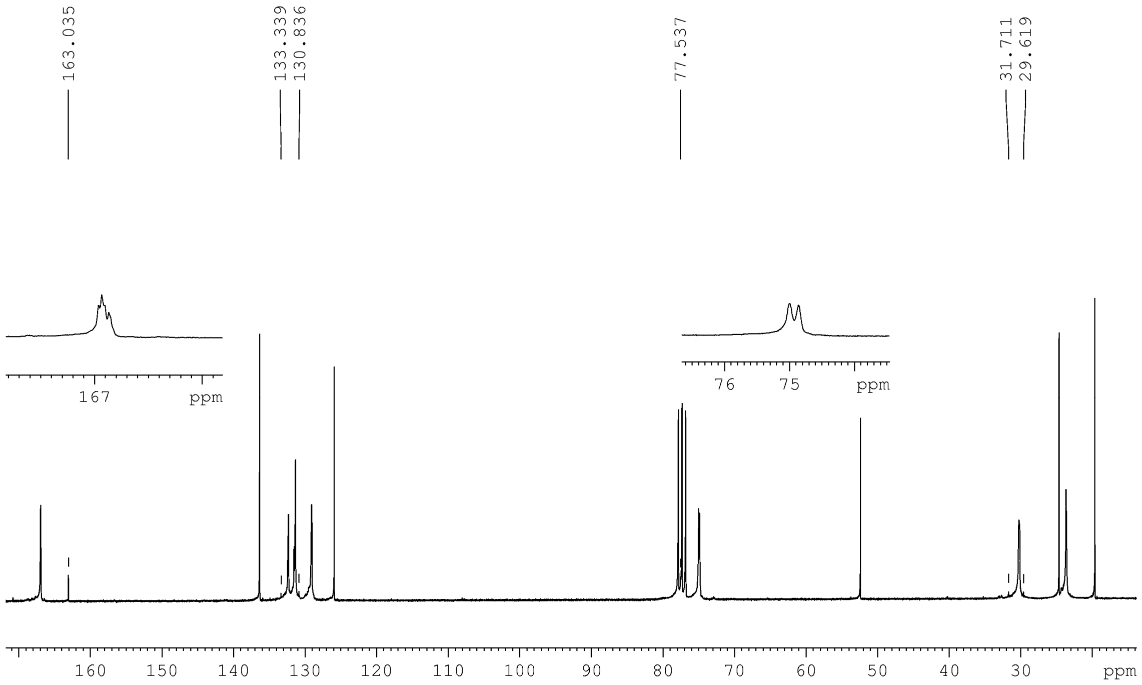

Figure S6. ^{13}C NMR spectrum (100 MHz, $CDCl_3$, 298K) with enlargements of the carbonyl region (left) and methine (right) regions of CHO/SA copolymer obtained in entry 6 of Table 1

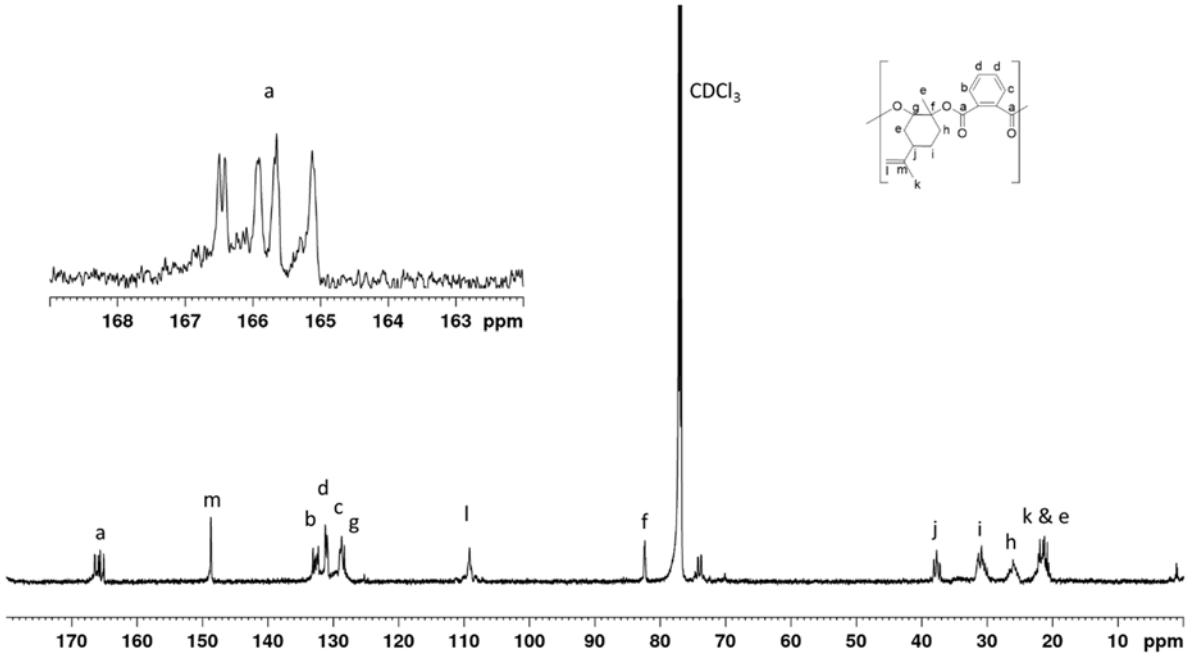

Figure S7. ^{13}C NMR spectrum (100 MHz, $CDCl_3$, 298K) with enlargements of the carbonyl region of LO/PA copolymer obtained in entry 5 of Table 1

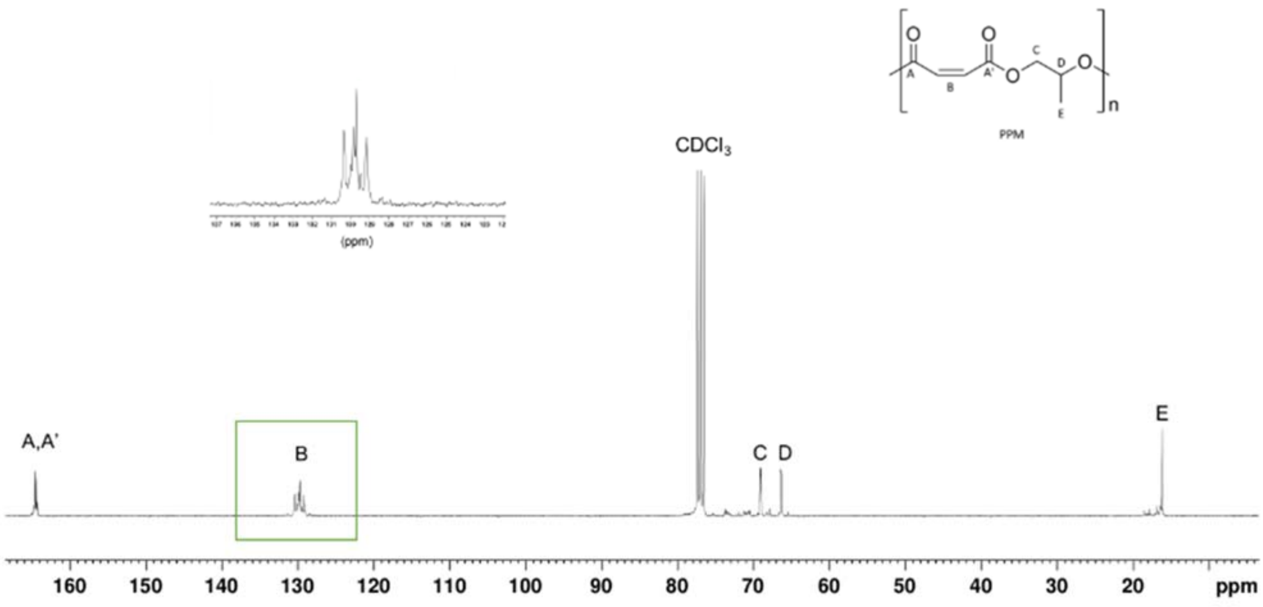

Figure S8. ^{13}C NMR (300 MHz, $CDCl_3$, 298 K) spectrum of poly(propylene maleate)

4. Gel permeation chromatography analysis (GPC)

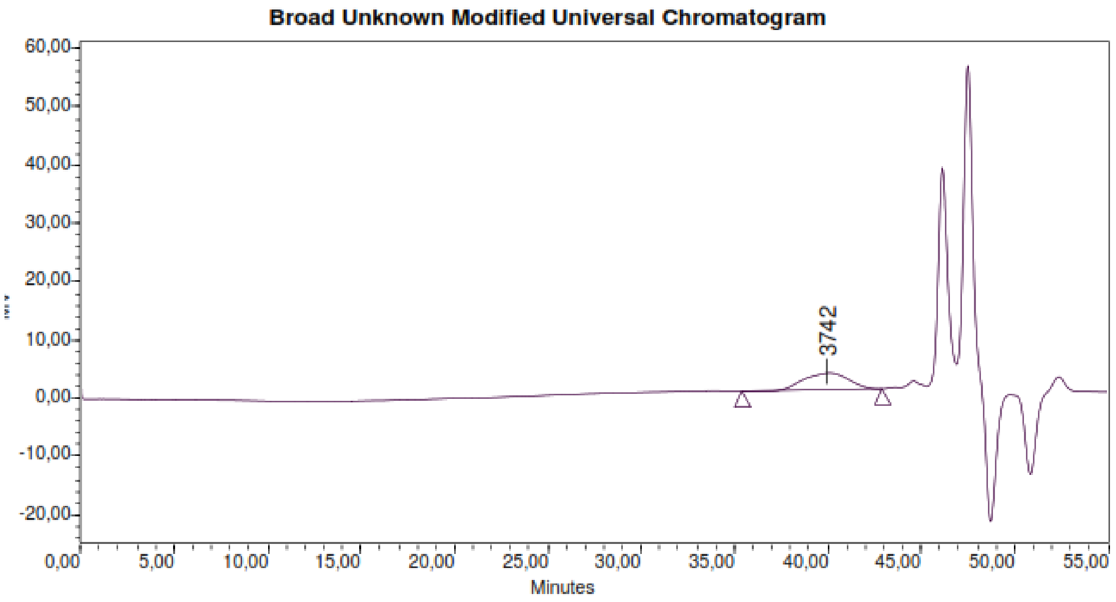

Figure S9. GPC of poly(propylene maleate)-block-polyglycolide
